# Auction-Based Secondary Relay Selection on Overlay Spectrum Sharing in Hybrid Satellite–Terrestrial Sensor Networks

**DOI:** 10.3390/s19225039

**Published:** 2019-11-19

**Authors:** Xiaokai Zhang, Bangning Zhang, Kang An, Zhuyun Chen, Daoxing Guo

**Affiliations:** 1 College of Communications Engineering, Army Engineering University, Nanjing 210007, China; xiaokaizhang@foxmail.com (X.Z.); zbnpub@163.com (B.Z.); chen_zhuyun@163.com (Z.C.); 2 The Sixty-Third Research Institute, National University of Defense Technology, Nanjing 210007, China; ankang89@nudt.edu.cn

**Keywords:** auction theory, hybrid satellite–terrestrial sensor networks (HSTSNs), relay selection, decode-and-forward (DF), amplify-and-forward(AF)

## Abstract

In this paper, we investigate the auction-based secondary relay selection on overlay spectrum sharing in hybrid satellite–terrestrial sensor networks (HSTSNs), where both the decode-and-forward (DF) and amplify-and-forward (AF) relay protocols are analyzed based on time division multiple access (TDMA). As both the primary and secondary networks are rational, honest but with incomplete network information, they prefer to obtain maximum possibility payoffs by the cooperation between the primary and secondary networks, and the competition among secondary networks. Hence, Vickery auction is introduced to achieve the effective and efficient secondary relay selection by distinct sub-time slot allocation for one shot in terms of a distributed manner. Finally, numerical simulations are provided to validate the effectiveness of the auction mechanism on cooperative spectrum sharing in HSTSNs for secondary relay selection. Besides, the effect of key factors on the performance of the auction mechanism are analyzed in details.

## 1. Introduction

Sensor networks are important enablers in a wide range of applications, such as the Internet of Things (IoT), environmental sensing, remote health monitoring, and environmental monitoring [1,2]. Massive Machine-type communications (mMTC) are classified as an identical scenario in 5G by the International Telecommunications Union (ITU) [3], where sensor networks are seen as typical applications. Furthermore, 5G mobile communication systems are anticipated to integrate various radio access technologies, including the satellite, space and aerial component [4,5]. 3GPP has recently started on Non-Terrestrial Networks (NTN) for 5G systems [6], which applies the satellite to support the terrestrial un-served area (isolated/remote areas, onboard aircraft or vessels) and underserved areas (e.g., suburban/rural areas) [7,8,9,10]. In most applied sensor scenarios, sensors are distributed over a wide range area, in some cases, they are located in remote areas and cannot be served by the terrestrial networks [11]. Also, the satellite segment significantly reduced vulnerability to physical attacks or natural disasters. Thus, the utilization of satellite systems becomes of paramount importance for the deployment of sensor networks [12], which improves service reliability thanks to a better service continuity for sensor nodes.

However, satellite sensor links mainly depend on the line-of-sight (LOS), which are vulnerable to be blocked by heavy shadowing or obstacles, such as buildings, bushes, etc., [13]. Besides, the unique features, such as low energy consumption, spectrum scarcity and implementation cost, limit the wide scope of usage. Fortunately, the quality of services (QoS) about terrestrial wireless sensor nodes can be augmented with the development of the relay within seamless connectivity and broadband access to mobile users [14]. To enhance coverage area and obtain highly reliable data rate services for satellite sensor networks, deployments of terrestrial relays in hybrid satellite–terrestrial sensor networks (HSTSNs) are proposed [15,16,17,18,19,20,21]. Various works have analyzed the performance of the HSTSNs by using the decode-and-forward (DF) and amplify-and-forward (AF) relay protocols [22,23,24]. On the other hand, the terrestrial networks are suffering from the spectrum scarcity. One of the available solutions is the cognitive radio (CR) in the hybrid framework, including interweave, underlay and overlay paradigm [25,26,27]. Inspired by the combination of CR and HSTSNs, the author in [28] firstly proposed the overlay paradigm in hybrid satellite–terrestrial networks (HSTNs), in which the secondary terrestrial users assist the primary satellite transmission through cooperative relaying techniques in exchange for spectrum access. Besides, the relay selection scheme is based on globe channel state information (CSI) from the perspective of the statistical channel model, where overheads include a huge time delay and the calculated amount for the onboard processing satellite or ground network control center (NCC). To further increase the system capacity, the author in [29] combined non-orthogonal multiple access (NOMA) and the overlay paradigm HSTNs. The works in [30,31] investigated the overlay paradigm into the holistic 5G system. However, those two works mainly focus on the terrestrial network, which is unable to apply in the HSTNs directly.

In this paper, we investigate the Vickery auction-based secondary relay selection for cooperative overlay spectrum sharing in HSTSNs. Especially, the potential relay would be the base-stations (BS) of the 5G, device-to-device (D2D) nodes or other ad-hoc networks, which are capable of applying the unlicensed frequency band. The main contributions of this paper are summarized as follows:

- First of all, we analyze both the DF and AF relay protocols on the spectrum sharing mechanism for primary user’s (PU’s) message in HSTSNs. In addition, the advantages and disadvantages of the traditional relay selection algorithm are also analyzed.
- Then, the Vickery auction mechanism is introduced to achieve the efficient and fairness secondary relay selection by one shot in a distributed manner, where the bids of the potential relay are the assistance transmission capacity by the different sub-time slot allocation in the entire time slot.
- Numerical simulations are provided to compare the proposed auction-based algorithm with the maximum satellite-relay and relay-destination link and maximum satellite-relay link, which validate the effectiveness of the auction mechanism on cooperative spectrum sharing in HSTSNs for secondary relay selection. Besides, the effects of key factors on the performance of the auction mechanism are analyzed.

The rest of this paper is organized as follows. Some related work is introduced in the Section 2. Section 3 presents the overlay spectrum sharing HSTSNs system model, analyzes the transmission capacity for AF and DF relay protocols. In Section 4, the Vickery auction mechanism is introduced. Section 5 shows the simulation results. Finally, conclusions are drawn in Section 6.

## 2. Related Works

Given the scenarios of multiple potential secondary relays, the primary sensor network tends to choose an optimal secondary network to cooperate for obtaining the maximum possible payoffs. In [32], the authors proposed to select the relay based on the knowledge of the instantaneous signal-to-noise ratios (SNRs) of the satellite-relay and relay-destination links. For practical application of such a complex scheme, CSI of all relay-destination links and satellite-relay is required at the satellite, which is provided by using feedback from the destination to the satellite. Due to the high latency in satellite systems, it is very difficult to provide CSI of all links at the satellite. For an alternative relay selection scheme, the satellite only requires the CSI feedback of the satellite-relay links from the relays [33], where the total capacity maximization can not be guaranteed. Besides, for the secondary networks, it is possible to increase network throughput through cooperative spectrum sharing. However, there exist required price paid, including the cost of booting energy consumption, hardware price, time-window value, etc. Besides, both primary and secondary networks are with incomplete information. Without considering the fairness or QoS assurance in cooperative overlay spectrum sharing networks, the secondary relay has no willingness to cooperate with the primary networks.

Market-driven mechanism consisted of auction-based or pricing-based approach can efficiently redeploy the scarce resource, balance the demands and even attract potential participants [34,35]. Auction theory received considerable attention for solving the problem of maximizing utilization in the case of resource scarcity, which is with uncertainty about the peculiar information and achieving competitive fairness [36]. The authors in [37,38] proposed auction-based resource allocation and relay selection in cooperative communications. However, to our knowledge, few related work is available for the satellite systems. Due to the long-time delay attributes of satellite communications, sequential auction mechanisms, such as English Auction and Dutch Auction, with uncertain bidding episodes, are improper for this scenario. Besides, since the satellite computational resource is limited, it is arduous to process excessive information in a centric manner. The sealed-bid second-price auctions, also named Vickery auctions, in which the bidder who submitted the highest bid is awarded the object being sold and pays a price equal to the second-highest amount bid, is that bidding one’s true value is a dominant strategy, which gives unique Nash equilibrium (NE) [39,40]. Hence, this mechanism can achieve efficient and effective decision by one shot in a distributed manner, which reduces considerable overhead including the decision time and satellite side computational complexity. The distributed manner here means that all potential relays make decision themselves by evaluating the true value of the cooperative spectrum sharing. Hence, we study auction-based relay selection on cooperation spectrum sharing in HSTSNs.

## 3. System Model

**Figure 1:**
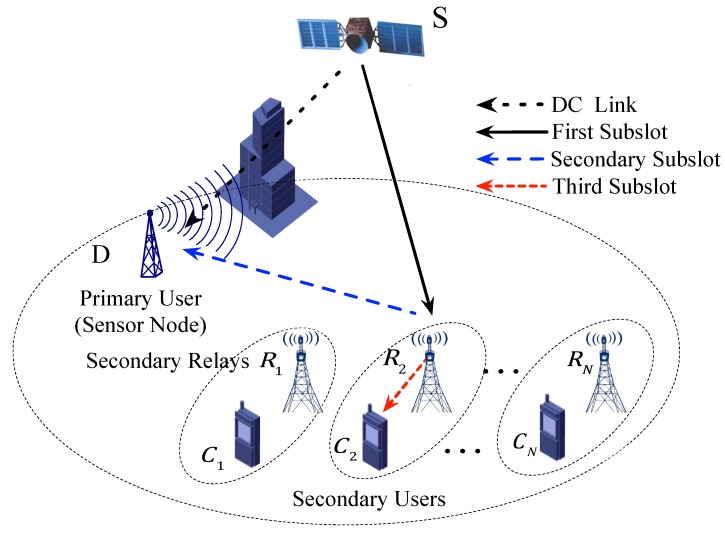
System model.

An overlay diagram of the HSTSNs with multiple potential secondary relays is considered as illustrated in Figure 1. This scenario comprises a primary satellite transmitter *S*, a sensor node *D*, i.e., the PU, potential CR secondary relays sets $\left\{R_{1},R_{2},\cdots ,R_{N}\right\}$ and the secondary users (SUs) sets $\left\{C_{1},C_{2},\cdots ,C_{N}\right\}$, respectively. Due to the heavy shadowing and obstacles, it is difficult to maintain a reliable direct communication (DC) link between *S* and *D*. Therefore, the primary satellite has to recruit a secondary transmitter as a relay. In return, the primary networks authorize secondary transmitter spectrum access opportunities. All this potential secondary relay would have the willingness to be recruited by the primary satellite network. The chosen secondary relay does its best to transmit the data as much as possible. Without loss of generality, we assume one PU and one SU to simplify the analysis, which can be easy to extend to multi-users scenarios by using other subchannels.

In the proposed model, all users access the channel based on time division multiple access (TDMA) to transmit their data, which means that the time slot is divided into three sub-time slots. The first fraction of a time slot is used for primary satellite transmitter broadcast its data to the selected secondary relay, and then, the selected relay retransmits the signal to the PU in the secondary sub-time slot by AF or DF relay protocols. At the last sub-time slot, the selected relay transmits its signal to the PU as payoffs. Therefore, if the multi sensor nodes are exist, the same progress can implement in different subchannels.

### 3.1. Sub-Time Slot Analysis

In this paper, we assume all channels follow quasi-static fading, i.e., the channel gains remain to be constant within each transmission block. The auction process is applied in every block interval. The secondary relays can get instantaneous CSI between two receivers by the pilot, and the relays don’t share the CSI among them. Therefore, potential relays are with incomplete information. One of the main advantages is the reduction of the overhead of CSI interaction. We assume the primary network selects the $R_{k}$ as the cooperative relay, where $R_{k}\in \left\{R_{1},R_{2},\cdots ,R_{N}\right\}$. $H_{XY}$ denotes the channel between X and Y, where X, Y could be *S*, $R_{k}$, *D*, and $C_{k}$, respectively. The channel power gain is written as $G_{XY}={\left|H_{XY}\right|}^{2}$. Furthermore, if the satellite *S* chooses $R_{k}$ as a relay, it indicates that $H_{SR_{k}}$ is always better than $H_{SD}$. For the assistance of the primary satellite communication, the secondary relay chooses DF or AF relay protocols for the transmission. In the considered system, each node has a single antenna, working in a half-duplex mode (which means that the node cannot transmit and receive simultaneously).

In this paper, $x_{p}$ and $x_{k}$ denotes the signal of satellite transmitter and the *k*th relay respectively, where $E\left[{\left|x_{p}\right|}^{2}\right]=E\left[{\left|x_{k}\right|}^{2}\right]=1$, and $E\left[\cdot \right]$ means the expectation operation. The satellite sends the signal to the cognitive relay in the first sub-time slot, which can be expressed as

(1) $$y_{SR_{k}}=\sqrt{P_{S}}H_{SR_{k}}x_{p}+n_{0},$$

where $P_{S}$ is the satellite transmitter power, and $n_{0}$ is the additive white Gaussian noise (AWGN) with mean zero and variance $\sigma_{0}^{2}$. For the second sub-time slot, the chosen relay could choose the DF or AF relay protocols. For DF relay protocols, the received signal of PU is given by

(2) $$y_{R_{k}D}=\sqrt{P_{k}}H_{R_{k}D}x_{p}+n_{1},$$

where $P_{k}$ is the transmitter power of $R_{k}$, we assume the same AWGN noise power is $\sigma_{1}^{2}$. For AF relay protocols, the second sub-time slot received signal of PU is

(3) $$y_{R_{k}D}=\sqrt{P_{k}}\frac{H_{R_{k}D}y_{SR_{k}}}{\sqrt{{\left|y_{SR_{k}}\right|}^{2}}}+n_{1}.$$

In the third sub-time slot, the chosen relay would transmit own signal to the SU, the received signal is given by

(4) $$y_{R_{k}C_{k}}=\sqrt{P_{k}}H_{R_{k}n}x_{k}+n_{2},$$

where the AWGN noise power is $\sigma_{2}^{2}$.

### 3.2. Transmission Capacity

**Figure 2:**
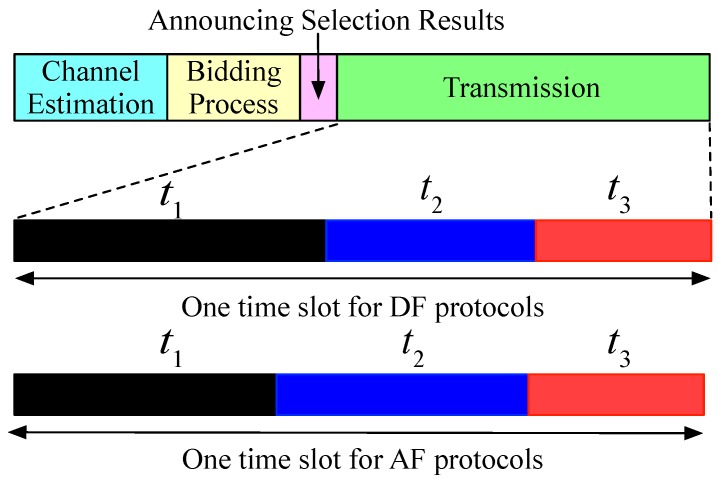
Time frame structure for auction-based selection and time slot design for DF and AF relay protocols.

As we can see, if the cognitive relay is decided to assist to retransmit the primary signal, some price is needed to pay, such as the cost of booting energy consumption, hardware price, time-window value, etc. Hence, we assume that the secondary network needs to achieve the SU’s QoS requirements, i.e., the minimum requirement transmits rate $R_{\min}$, which is obtained by the third sub-time slot $\left(t_{3}\right)$. The total duration of the time slot is *t*. The total frame and time slot allocations are shown in Figure 2.

For AF relay protocols, the duration of first and second sub-time slot are the same, i.e., $t_{1}=t_{2}$. Therefore, the capacity of SU is given by

(5) $$R_{AF_{k}}\left(t_{2}\right)=t_{2}\log_{2}\left(1+\frac{\gamma_{1}\gamma_{2}}{\gamma_{1}+\gamma_{2}+1}\right).$$

The transmitted capacity of third sub-time slot is given by

(6) $$R_{R_{k}C_{k}}\left(t_{3}\right)=t_{3}\log_{2}\left(1+\gamma_{3}\right),$$

where γ3=PkGRkCkPkGRkCkσ22σ22.

For DF relay protocols in the first two temporal sub-time slots, i.e., $t_{1},t_{2}$, the transmitted capacity is given by

(7) $$R_{DF_{k}}\left(t_{1},t_{2}\right)=\min\left[t_{1}\log_{2}\left(1+\gamma_{1}\right),t_{2}\log_{2}\left(1+\gamma_{2}\right)\right],$$

where γ1=PSGSRkPSGSRkσ02σ02, γ2=PkGRkDPkGRkDσ12σ12.

According to the above analysis, the proper secondary relay selection is key to obtaining both the enhanced primary capacity and sum capacity. There are two conventional schemes about relay selection. The first one is that the satellite can select a relay based on the knowledge of the instantaneous SNRs of the satellite-relay and relay-destination links, which can select the best relay theoretically. For practical application of such a complex scheme, CSI of all relay-destination and satellite-relay links is required at the satellite, which is provided by using feedback from the destination to the satellite. Due to the high latency in satellite systems, it is very difficult to provide CSI of all links at the satellite. Besides, the computational complexity is also quite higher for on-board processing satellite. Another simple relay selection scheme is that the satellite only requires the CSI feedback of the satellite-relay links from the relays, where the total capacity maximization can not be guaranteed. Those two schemes are centralized ways. Actually, the relays are aware of the CSIs of both relay-destination and satellite-relay links and the relays are isolated. In the overlay paradigm, the relays are competed with each other. If a proper scheme is implemented, the relays could tell the true ability in a distributed manner and the whole systems obtain the maximum capacity.

## 4. Auction Game

In this section, we implement the auction theory to investigate the interaction among potential relays, where the best relay can be selected by one shot in a distributed manner. The distributed manner here means that all potential relays make decision themselves by evaluating the true value of the cooperative spectrum sharing.

### 4.1. Auction Mechanism Selection

The auction game provides a specific set of rules that will govern the sale or purchase of an object to the submitter of the most favorable bid. Therefore, the auction mechanism is exactly suitable for scenarios where resources are used to their full potential. In view of the long-time delay attributes of satellite communications, sequential auction mechanisms, such as English Auction and Dutch Auction, are not suitable for this scenario because of uncertain bidding episodes.

Furthermore, since the satellite computational resource is extremely limited, it is hard to process too much information in a centric manner. There are two popular sealed-bid auction mechanisms including first-price auctions and second-price auctions. In a first-price auction, the bidder who submitted the highest bid is awarded the object being sold and pays a price equal to the amount of the bid. Since the payment is equal to the amount bid, bidders shade their bids below their true value. when combining sealed-bid and first-price, it is hard to reach a NE. For the sealed-bid second-price auction, bidding one’s true value is a dominant strategy, which gives unique NE. Thus, this mechanism can achieve efficient and effective decision making by one shot in a distributed manner, i.e., the satellite select the biggest bidder as the chosen relay, which reduce the decision time and computational complexity for onboard processing satellite or NCC of the ground getaway.

We assume that all the secondary and primary networks are rational, honest but with incomplete global information, and they expect maximum payoffs from the cooperation spectrum sharing structure. Since the multi-relay cooperation at the same time is overhead in synchronization and coding, we assume the primary satellite system only recruits the best relay to retransmit the signal. We choose the Vickery auction mechanism for the relay selection by one shot in multi-relay HSTSNs. All potential relays are the bidders, who must compete with each other for the opportunity spectrum sharing. The satellite is the auctioneer, who would choose the best bids and broadcast all bids and the results to all bidders.

### 4.2. Bidder Private Values

The author in [29] gives a monetary as bids in wireless communications of the auction game. However, it is laborious to perceive the monetary coefficient in an authentic scenario. Due to the characteristics of mutual aid in the overlay paradigm, the value of potential relay for the satellite is the assistance transmitted capacity. Therefore, the assistance transmitted capacity is the bid for the potential relay, which can be calculated in each relay. Besides, due to the cost of booting energy consumption, hardware price, time-window value and etc, the bidders require the minimum transmission rate $R_{\min}$, which is the lowest QoS requirement of the secondary network. Otherwise, relays have no willingness to join the network for cooperation.

The winner of the Vickery auction is the one who offers the highest price and pays the amount of the highest non-winning bids. Consequently, the dominant strategy for each bidder is to transmit the remaining power to the primary user under the requirement of meeting the $R_{\min}$. Forward, the minimum third sub-time slot is given as

(8) $$t_{3-bid}=R_{R_{k}C_{k}}^{-1}\left(R_{\min}\right),$$

where the superscript ${}^{-1}$ is the reverse function. For AF relay protocols, the first two sub-time slots can be given as t1=t2=t−t3t−t322. The bids for $R_{k}$ is

(9) RAFk−bids=RAFkt3−bidt−t322,

where $R_{AF_{k}}\left(*\right)$ can be calculated by (5). In order to obtain the maximum bids for DF relay protocols, the relay has to reconfigure the first two sub-time slots. If the first sub-time slot received signal can be transmitted to the PU, the PU obtain the maximum capacity. Therefore, we have

(10) $$t_{1-bids}\log_{2}\left(1+\gamma_{1}\right)={t_{2}}_{-bids}\log_{2}\left(1+\gamma_{2}\right).$$

In addition, $t_{1-bids}+t_{2-bids}=t-t_{3-bids}$. Hence, the bid for DF relay protocol is given as

(11) $${R_{DF_{k}}}_{-bids}=R_{DF_{k}}\left(t_{1-bids},t_{2-bids}\right),$$

where ${R_{DF_{k}}}_{-bids}\left(*\right)$ can be calculated by (7).

### 4.3. Auction Design

From a cooperative perspective, the optimal relay here is the one that provides the most benefit to the source transmission under the relay QoS constraint. Vickery auction has the advantage of assigning the item for sale to the bidder who values it most.

The Vickery auction can be described as follows:

- *Information:* We assume all potential relays could use the maximum power $P_{k}$ to achieve as much capacity as possible. The publicly available information includes the noise density $\sigma_{0}^{2}$ and $\sigma_{1}^{2}$. Each relay, acted as a bidder, could acquire the channel gain $G_{R_{k}C_{k}}$, $G_{R_{k}D}$ and $G_{SR_{k}}$, which is not available for other bidders. Hence, the incomplete information prevent bidders from lying, and each bidder would offer the true value for utilizing the shared spectrum.
- *Bids:* The value of potential relay for the satellite is the assistance transmitted capacity. All potential relay would send the bids ${R_{AF_{k}}}_{-bids}$ or ${R_{DF_{k}}}_{-bids}$ to the satellite.
- *Allocation:* The satellite chooses the highest bids as the winner. In addition, the satellite broadcasts all the bids to all potential relays and informs the selected secondary relay the highest non-winning bids. Once the satellite is lying, all potential relay quit as a penalty. The potential Relays are physically isolated from each other, so avoiding collusion.
- *Payoffs:* It can be aware of that the PU obtain the highest non-winning bids $R_{pri}$ as payoffs. The chosen relay $R_{k}$ reconfigures the sub-time slots allocation by the highest non-winning bids. For AF relay protocols, the first two sub-time slots are reconfigured as
  (12) $${t^{\prime }}_{1}={t^{\prime }}_{2}=R_{AF_{k}}^{-1}\left(R_{pri}\right).$$
  For DF relay protocols, the first two sub-time slots are reconfigured as
  (13) $$\left({t^{\prime }}_{1},{t^{\prime }}_{2}\right)=R_{DF_{k}}^{-1}\left(R_{pri}\right).$$
  In addition, the first two sub-time slots still obey (10), which means the ${t^{\prime }}_{1},{t^{\prime }}_{2}$ can be obtained. The third sub-time slot is ${t^{\prime }}_{3}=t-{t^{\prime }}_{1}-{t^{\prime }}_{2}$. The payoffs of the relay is $R_{R_{k}C_{k}}\left({t^{\prime }}_{3}\right)$ After the sub-time slot reconfiguration, the chosen relay informs the satellite the time slot configuration and starts the transmission. It is worth to note that if there are bidders with the same bid, the primary network would randomly select one as the cognitive relay, and the payment will be paid at the highest price.

According to the auction mechanism proposed for relay selection and the system assumptions, the truthful bidding is a NE of the Vickery auction for the relay selection in a distributed manner.

It can be seen from the above analysis that the satellite does not need to obtain the global CSI, and the calculation complexity that needs to be paid is to select the highest one from all the bids. Therefore, the proposed relay selection mechanism has a low complexity.

Besides, the proposed auction-based mechanism makes sure the minimum transmission QoS requirements, i.e., $R_{min}$, which is the embodiment of fairness and facilitates the completion of the cooperation of primary network and the secondary network. The design of this mechanism guarantees the long-term cooperation.

## 5. Simulation Results

In this section, representative simulation results are presented to evaluate the effectiveness of the auction-based mechanism and the impacts of key factors on the performance of the proposed scheme. For the link between the satellite and the relay $R_{k}$, the channel gain is modeled as $G_{SR_{k}}=L_{SR_{k}}G_{r}G_{s}h_{SR_{k}}^{2}$, where $L_{SR_{k}}$ is the free space loss, $G_{r}$ and $G_{s}$ are the terrestrial receiver and satellite transmitter antenna gains, respectively, $h_{SR_{k}}$ is the small scale of fading, undergoing the Shadowed-Rice distribution [41,42,43]. For the ground link, the channel gain is modeled as $G_{R_{k}n}=d_{R_{k}n}^{-\eta }h_{R_{k}n}$, where *d* is the distance between $R_{k}$ and *n*, *n* can be *D* or $C_{k}$, η is the path loss exponent, $h_{R_{k}n}$ is the small scale of fading, undergoing the Rayleigh distribution [44]. Detailed system parameters are provided in Table 1.

**Figure 3:**
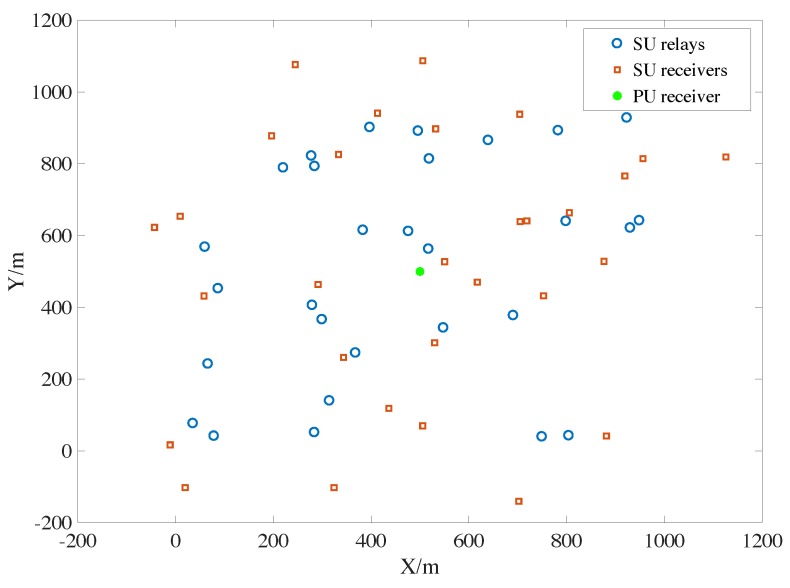
Uniform random distribution model in the cases of urban and populated scenarios.

We assume the secondary relays follow the uniform distribution around the primary user in square range with the side length of 1000 m. The secondary receiver obeys uniform distribution around its transmitter in a circle with a radius of 300 m. We generate 30 relays and the users, respectively, for example in Figure 3.

**Figure 4:**
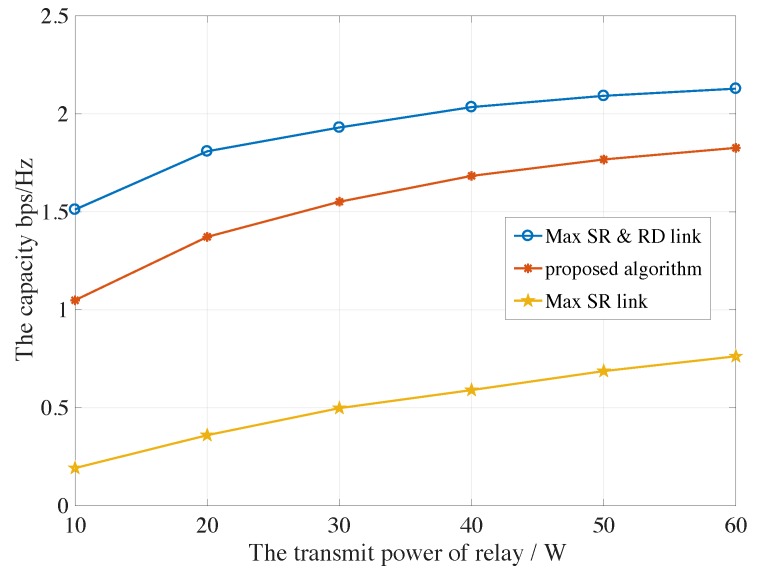
The capacity of PU under the maximum satellite-relay and relay-destination scheme, the proposed algorithm and maximum satellite-relay scheme verse different transmit power of relays for AF protocols.

**Figure 5:**
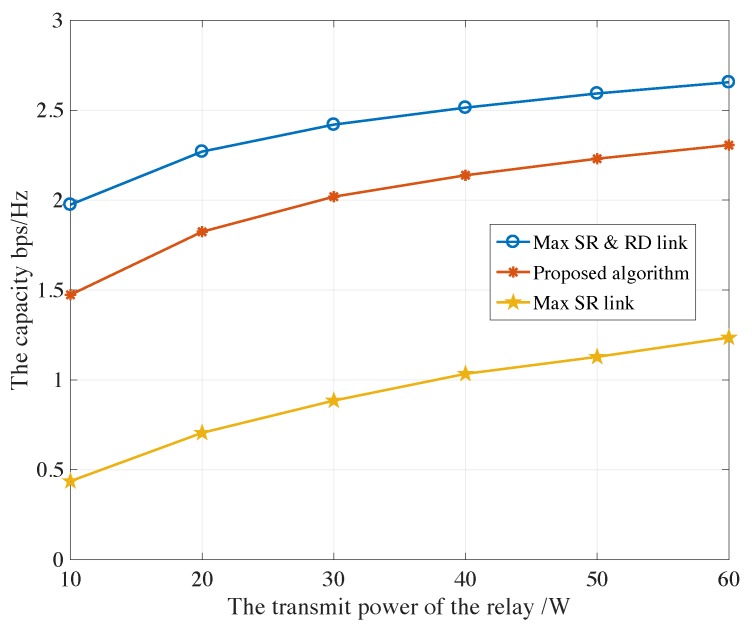
The capacity of PU under the maximum satellite-relay and relay-destination scheme, the proposed algorithm and maximum satellite-relay scheme verse different transmit power of relays for DF protocols.

Figure 4 and Figure 5 reveal the capacity of PU under the maximum satellite-relay and relay-destination scheme, the proposed auction-based algorithm and maximum satellite-relay scheme verse transmit power of relays $P_{k}$ from 10 W to 60 W for AF and DF protocols, respectively. Besides, all selected the secondary networks are at least satisfied the $R_{min}$. The optimal capacity is achieved for the maximum satellite-relay and relay-destination scheme, which acquires the globe CSIs and can be seen as the upper boundary, but the overheads are always beyond the satellite processing capability, which would cause large delay. The capacity of PU for proposed auction-based algorithm is slightly lower than the maximum satellite-relay and relay-destination scheme, because the partial CSI is implemented by relay, and the SU obtains more capacity. Although the computation consumption is the same as the maximum satellite-relay link scheme for satellite, a significantly improved capacity can be realized.

**Figure 6:**
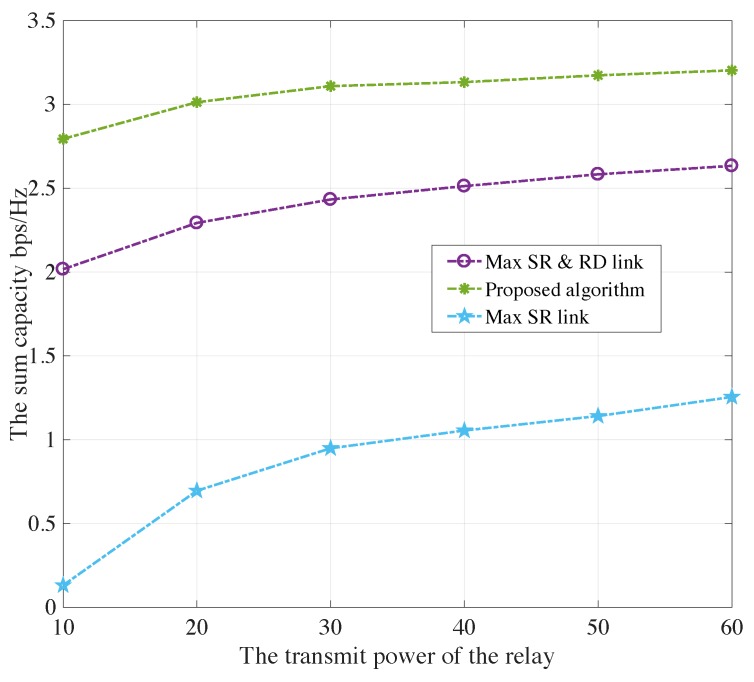
The sum capacity of both PU and SU under the maximum satellite-relay and relay-destination scheme, the proposed algorithm and maximum satellite-relay scheme verse different transmit power of relays for AF protocols.

**Figure 7:**
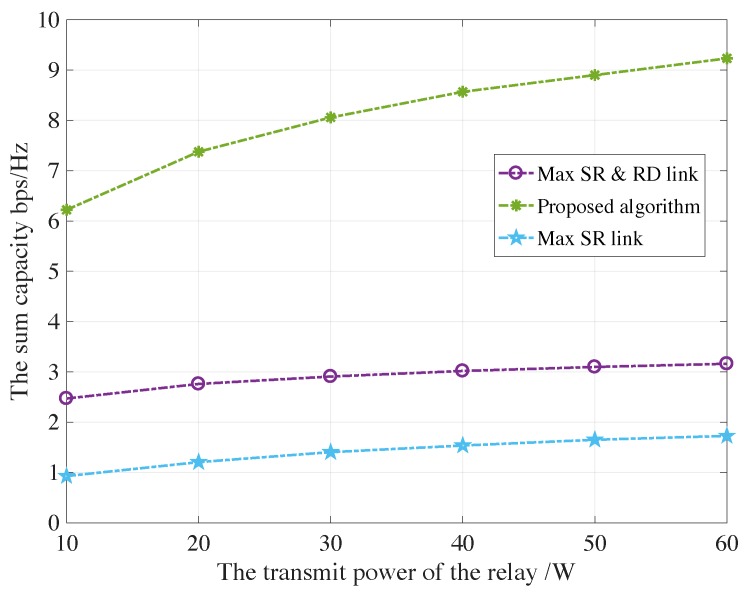
The sum capacity of both PU and SU under the maximum satellite-relay and relay-destination scheme, the proposed algorithm and maximum satellite-relay scheme verse different transmit power of relays for DF protocols.

Figure 6 and Figure 7 show the sum capacity of both PU and SU under the maximum satellite-relay and relay-destination scheme, the proposed auction-based algorithm and maximum satellite-relay scheme verse transmit power of relays $P_{k}$ from 10 W to 60 W for AF and DF protocols, respectively. The proposed algorithm obtain much more sum capacity than conventional schemes, which is achieved by the auction mechanism for the maximum possible capacity of secondary network. Besides, the satellite avoids global CSI acquisition and large computational complexity. Those facts indicate that the proposed auction-based relay selection scheme is effective, leading to a win-win situation for both the primary network and the secondary network.

**Figure 8:**
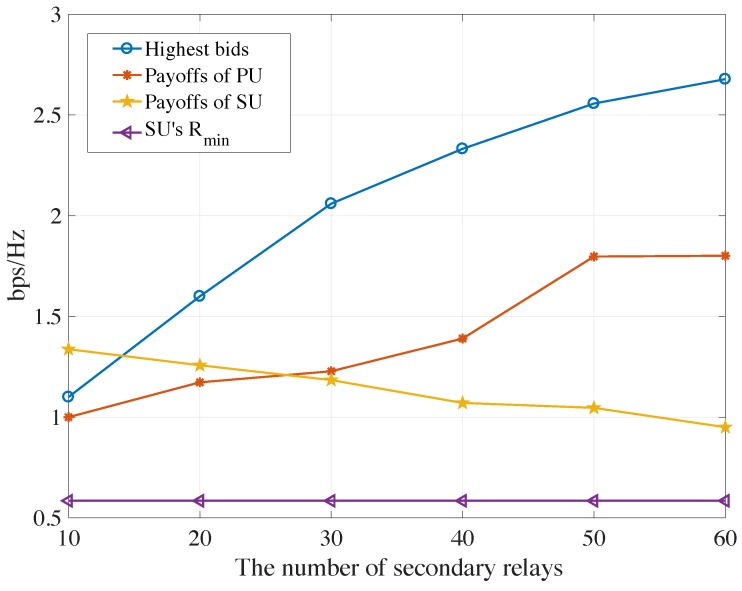
The highest bids, payoffs of PU and SU and $R_{min}$ verse numbers of potential relays for AF protocols.

**Figure 9:**
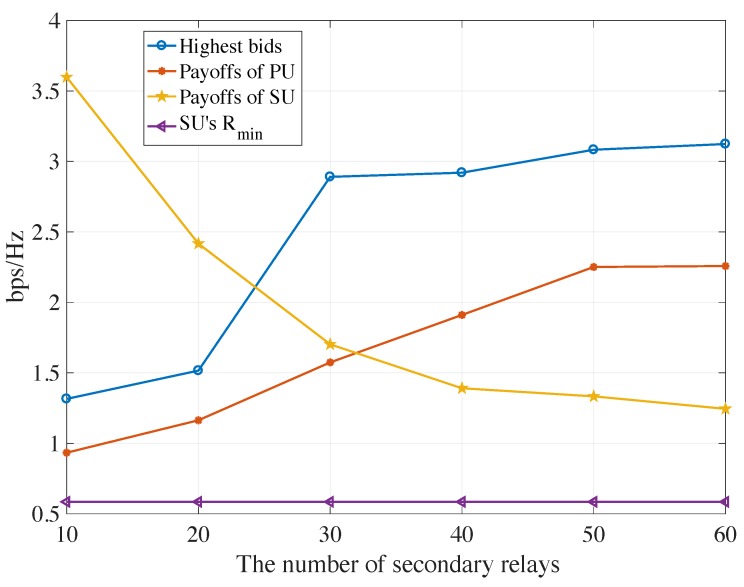
The highest bids, payoffs of PU and SU and $R_{min}$ verse numbers of potential relays for DF protocols.

Figure 8 reveals the highest bids, payoffs of the PU and SU in AF protocols, valued by the channel capacity, where the secondary relays’ transmitted power and AWGN power is 10 W and −40 dBw, respectively. It is observed that the payoffs of the PU increase as the number of secondary relays increases. However, payoffs of the chosen SU continue to decrease as the number of potential competitors increases, which is the same as the market place. If the primary would like to obtain as many payoffs as possible, It must recruit as many secondary relays as possible for the competition. In some extent, the highest bid is the up-boundary of the capacity of the PU and the minimum requirement of the SU $R_{min}$ is the low-boundary of the capacity of the SU. Figure 9 illustrates the highest bids, payoffs of the primary and secondary users in DF protocols, where the same trends can be seen as shown in the AF mode.

**Figure 10:**
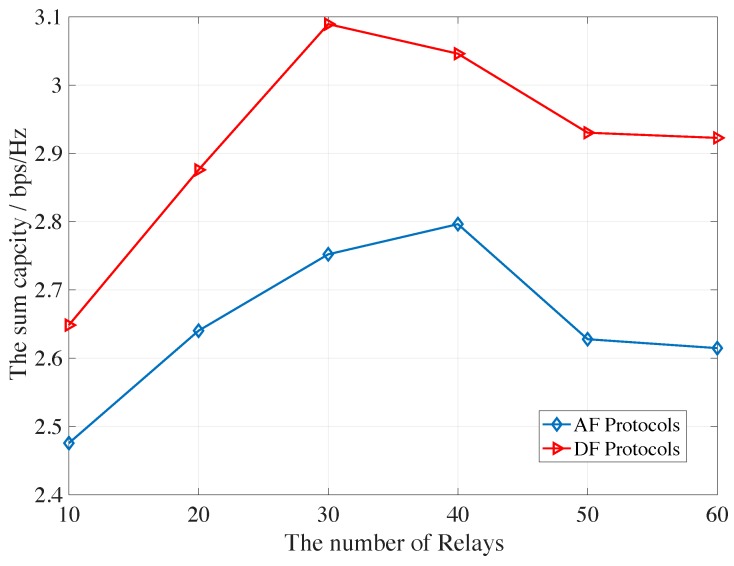
The total capacity for the chosen relays verse numbers of potential relays for both AF and DF protocols.

Finally, Figure 10 illustrates the total transmitted capacity for both the primary network and the secondary network versus the different number of the potential relays. It is obvious that payoffs of the AF protocol are lower than that of the DF protocol, which is due to the fact that the noise is amplified from the first temporal phase for the AF relay protocol. However, the DF relay protocol is more complicated.

## 6. Conclusions

In this paper, we investigate the Vickery auction-based secondary relay selection on a cooperative overlay spectrum sharing in HSTSNs. We analyze both the DF and AF relay protocols on the spectrum sharing mechanism for PU’s message by TDMA, which is built on the basis of the auction mechanism. Then, the Vickery auction mechanism is introduced to achieve efficient and fairness secondary relay selection by one shot in a distributed manner, where the bid of the potential relay is the assistance transmission capacity by the different sub-time slot allocation in the entire time slot. The simulation results show the efficiency of the Vickery auction mechanism for relay selection in HSTSNs.

## Figures and Tables

**Table 1:** Simulation Parameters.

Parameters	Values
Satellite transmission power	100 w
AWGN power	−108 dBw
Satellite transimiter antenna gain $G_{s}$	20 dB
Relay receiver antenna gain $G_{r}$	25 dB
Center frequency	4 GHz
Nakagami fading parameter of $H_{SR_{k}}$	19.4
Scatter component of $H_{SR_{k}}$	0.158
Terrestrial path loss exponent	2
Number of realizations	$10^{4}$

## Abbreviations

The following abbreviations are used in this manuscript:

HSTSN	hybrid satellite–terrestrial sensor network
DF	decode-and-forward
AF	amplify-and-forward
TDMA	time division multiple access
IoT	Internet of Things
mMTC	Massive Machine-type communications
ITU	International Telecommunications Union
NTN	Non-Terrestrial Networks
LOS	line-of-sight
QoS	quality of services
CR	cognitive radio
CSI	channel state information
NCC	network control center
NOMA	non-orthogonal multiple access
SNR	signal-to-noise ratio
NE	Nash equilibrium
BS	base-stations
D2D	device-to-device
PU	primary user
SU	secondary user
DC	direct communication
FDMA	frequency division multiplexing access
AWGN	additive white Gaussian noise
LEO	Low Earth Orbit
